# Research Progress on the Structure–activity Relationship and Mechanism of Flavonoid Derivatives in the Treatment of Lung Cancer

**DOI:** 10.3390/molecules30081827

**Published:** 2025-04-18

**Authors:** Jiacheng Yao, Feng Zhu, Yikun Feng, Chen Gu, Tianyu Wang, Xinyu Li, Hao Yang, Xiamin Hu, Pierre-Antoine Bonnet, Xiangguo Meng

**Affiliations:** 1 Graduate School, Shanghai University of Traditional Chinese Medicine, Shanghai 201203, China; yaojiacheng010419@126.com (J.Y.); fengyikun57@gmail.com (Y.F.); guchen0911@gmail.com (C.G.); 2 Faculty of Pharmacy, Shanghai University of Medicine and Health Sciences, Shanghai 201318, China; 17779812779@163.com (T.W.); lixinyu050318@163.com (X.L.); y_tian_young@stu.sumhs.edu.cn (H.Y.); 3 Yangzijiang Pharmaceutical Group Shanghai Haini Pharmaceutical Co., Ltd., Shanghai 201318, China; wangheng@yangzijiang.com; 4 IBMM, Faculty of Pharmacy, Montpellier University, CNRS, ENSCM, 34093 Montpellier, France

**Keywords:** flavonoids, anti-tumor mechanism, structure–activity relationships, natural products, derivate, lung cancer

## Abstract

Non-small-cell lung cancer (NSCLC) is the leading cause of cancer-related deaths worldwide. The difficulty in early diagnosis, combined with the tendency for tumor invasion and metastasis, creates significant challenges for current therapeutic approaches. Additionally, the pharmaceutical agents currently used to treat NSCLC often come with severe side effects and can lead to drug resistance. As a result, there is an urgent need to develop new therapeutic agents with fewer side effects that can effectively overcome resistance mechanisms. Flavonoids, a prominent class of natural compounds, have shown promise in preventing and treating various cancers. By structurally optimizing flavonoids, it is possible to enhance their anticancer activity and improve their pharmacokinetic properties. This article reviews the different mechanisms of action and structure–activity relationships (SARs) of flavonoid derivatives in treating NSCLC, aiming to provide a scientific foundation for developing new therapeutic agents.

## 1. Introduction

Statistics from GLOBOCAN 2022 indicated that approximately 2.5 million new cases of lung cancer were diagnosed worldwide, accounting for 12.4% of all cancer cases, making it the most prevalent cancer type [1]. Among them, non-small-cell lung cancer (NSCLC) accounts for 80% to 85% of lung cancer cases [2]. Although progress has been made in treatment, the prognosis for lung cancer remains poor, primarily due to late-stage diagnoses [3]. Recent data show that lung cancer continues to be the leading cause of cancer-related mortality in China, with a higher incidence in men compared to women, likely due to higher smoking rates among males [4]. Projections suggest that by 2035, the incidence of lung cancer will continue to rise in most countries, with China expected to account for the highest number of new cases worldwide. This trend has led to a significant increase in the demand for new cancer treatment drugs. However, many anticancer drugs come with a range of side effects. For example, cisplatin can cause acute kidney injury, gastrointestinal disorders, bleeding, and immunosuppression [5]. Tyrosine kinase inhibitors (TKIs) are frequently linked to serious cardiac complications, including hypertension, atrial fibrillation, cardiac dysfunction, and heart failure [6]. As a result, researchers are striving to develop new cancer drugs and to improve existing ones to mitigate these side effects.

**Figure 1:**
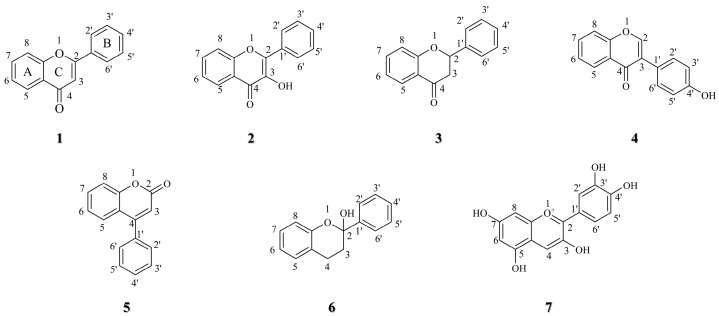
Structural types of flavonoids.

Natural products have played a crucial role in the history of anticancer drug development. Many key anticancer drugs, such as irinotecan, vincristine, etoposide, and paclitaxel, originate from plants, while actinomycin D and mitomycin C are derived from bacteria, and bleomycin comes from marine organisms. Among them, camptothecin and paclitaxel stand out as highly successful cases. Camptothecin exerts its anticancer effect by inhibiting topoisomerase I to interfere with DNA replication. Through structural modification, improved derivatives like irinotecan and topotecan have demonstrated enhanced solubility and reduced toxicity, subsequently becoming mainstays for treating various cancers, including ovarian and lung cancers [7]. Beyond classical plant-derived chemotherapeutics, flavonoids represent a structurally diversified class with privileged scaffolds for drug design. Natural flavonoids have been found to possess diverse pharmacological effects, including anti-inflammatory [8], antioxidant [9], hypoglycemic [10], hepatoprotective [11], nephroprotective [12], neuroprotective [13,14], and antiviral properties [15]. The flavonoid structure consists of two aromatic rings (A and B rings) and a heterocyclic ring (C ring), forming a 15-carbon C6-C3-C6 basic skeleton (compound **1**, Figure 1) [16]. Based on their chemical structure, oxidation state, and the unsaturation of the linked chains, flavonoids can be classified into the following several categories (compounds **1~7**, Figure 1): flavones (compound **1**), flavonols (compound **2**), flavanones (compound **3**), isoflavonoids (compound **4**), neoflavonoids (compound **5**), flavanols (compound **6**), and anthocyanins (compound **7**) [17]. Previous studies have demonstrated that natural flavonoids modulate apoptosis, angiogenesis, and the tumor microenvironment, making them promising candidates for lung cancer therapy [18,19,20,21]. Collectively, many reviews highlight the inexhaustible significance of flavonoid-based natural product structures in anti-lung cancer drug discovery and development [21,22,23]. Despite their therapeutic promise, the clinical application of natural flavonoids in anticancer therapy is hindered by intrinsic structural limitations, including low bioavailability, poor stability, inadequate solubility, inefficient targeted delivery, and drug resistance [24,25]. Structural modification has emerged as a key strategy to overcome the limitations of natural flavonoids and enhance their anti-tumor activity [26,27]. Synthetic flavonoid derivatives such as halogenated, glycosylated, or metal-complexed analogs exhibit enhanced pharmacological profiles, including improved anticancer efficacy and metabolic stability [28,29,30]. Based on the flavonoid skeleton and the proven properties against lung cancer, the isoflavone scaffold is a privileged structure for the development of anticancer drugs. This review summarizes the anti-lung cancer mechanisms and structure–activity relationships (SARs) of flavonoid derivatives and describes the mechanism of action of derivatives and the signaling pathways involved, demonstrating how chemical modifications not only overcome the limitations of natural flavonoids but also enhance their anti-tumor efficacy, providing a theoretical basis for developing optimized anticancer agents.

## 2. Anti-Lung Cancer Mechanism of Flavonoid Derivatives

### 2.1. Induction of Apoptosis

**Figure 2:**
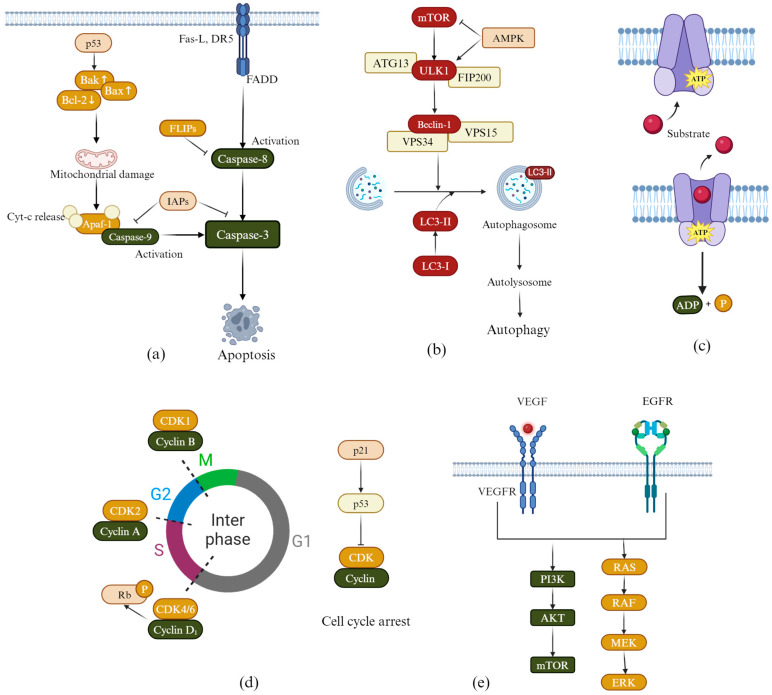
Anticancer mechanisms of flavonoid derivatives: (**a**) the mechanism of apoptosis, (**b**) the mechanism of autophagy, (**c**) the mechanism of overcoming multidrug resistance, (**d**) the mechanism of induction of cell cycle arrest, and (**e**) the mechanism of inhibiting tumor cell angiogenesis and inhibiting EGFR kinase activity.

The mechanisms of apoptosis are illustrated in Figure 2a. Cysteinyl aspartate-specific proteinases (Caspases) are key players in apoptosis. They induce apoptosis in lung cancer cells through both extrinsic and intrinsic pathways. In the extrinsic pathway, death receptor 5 (DR5) binds to Fas Ligand (FAS-L), which activates Caspase-8. This, in turn, activates Caspase-3, leading to apoptosis in lung cancer cells. FLICE inhibitory proteins (FLIPs) regulate this process by inhibiting Caspase-8. In the intrinsic pathway, the activation of the p53 protein leads to an increase in the following two pro-apoptotic factors: Bcl-2-associated protein X (Bax) and Bcl-2 homologous antagonist/killer (Bak). This increase enhances the permeability of the mitochondrial membrane, releasing cytochrome c (Cyt-c). Once released, Cyt-c will bind with apoptotic protease activating factor-1 (Apaf-1) to form apoptosomes. This process activates Caspase-9, subsequently activating Caspase-3, leading to apoptosis in lung cancer cells. B-cell lymphoma 2 (Bcl-2) protein prevents apoptosis by inhibiting the release of Cyt-c. Additionally, Inhibitors of Apoptosis Proteins (IAPs) further block apoptosis by inhibiting caspase activity [31,32].

**Figure 3:**
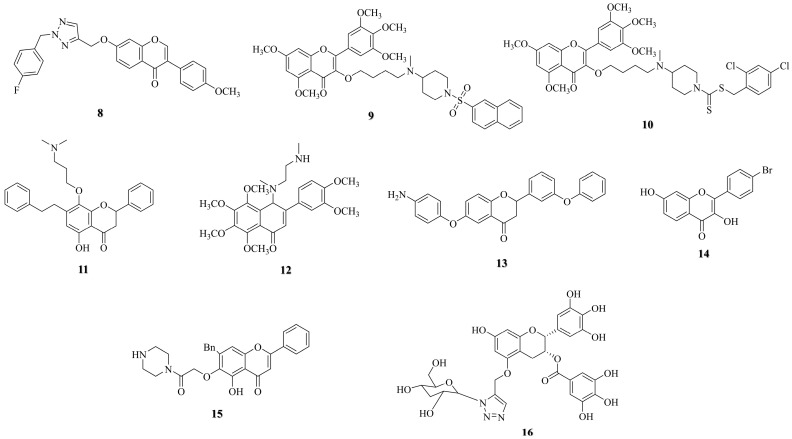
Structure of flavonoid derivatives.

Studies have shown that flavonoid derivatives can regulate the Bax/Bcl-2 ratio and induce apoptosis in lung cancer cells through endogenous pathways. For example, an isoflavone compound **8** (Figure 3) synthesized by Jia et al. [33] induced apoptosis in lung cancer cell line A549 by upregulating Bax and downregulating Bcl-2. The IC_50_ of flavonol compound **9** (Figure 3) synthesized by Li et al. [34] on A549 cells was determined to be 6.38 μM and 3.25 μM after 24 and 48 h, respectively. These compounds were found to activate Caspase-3 and p53. Similarly, another flavonol compound, **10** (Figure 3), developed by Zhou et al. [35], also promoted apoptosis in A549 cells through a related mechanism. In addition, compound **11** (Figure 3), which was synthesized by Bak et al. [36], induced apoptosis in the lung cancer cell lines NCI-H460 and A549 by upregulating Fas/FasL and activating the caspase cascade.

### 2.2. Induction of Autophagy

The mechanism of autophagy is illustrated in Figure 2b. The process begins with inhibition of the mammalian target of rapamycin (mTOR), which triggers activation of the Unc-51-like autophagy-activating kinase 1 (ULK1) complex, initiating autophagosome formation. AMP-activated protein kinase (AMPK) further promotes autophagy by simultaneously suppressing mTOR and activating ULK1. A key step in autophagosome maturation involves the Beclin-1–Vps34 complex, which mediates membrane nucleation, while microtubule-associated protein light chain 3 (LC3) serves as a critical marker. The conversion of cytosolic LC3-I to lipidated LC3-II reflects autophagosome maturation. Finally, autophagosomes fuse with lysosomes to form autolysosomes, where cellular components are degraded by lysosomal hydrolases, completing autophagy [37]. Given this pathway’s centrality in cancer, therapeutic strategies often target autophagy regulators such as AMPK/mTOR signaling, ULK1, and Beclin-1. For example, Li et al. [38] developed flavonoid compound **12** (Figure 3), which exhibited IC_50_ values of 3.2 ± 1.2 µM, 6.7 ± 0.9 µM, 10.2 ± 2.3 µM, and 9.8 ± 1.6 µM against the lung cancer cell lines CL1-5, H1299, H226, and A549, respectively. Mechanistic studies have demonstrated that compound **12** activates autophagy by triggering autophagosome formation and increasing LC3-II levels.

### 2.3. Overcoming Multidrug Resistance

Multidrug resistance (MDR) is a major challenge in treating tumors and is often seen as a significant factor in treatment failure. The mechanism of MDR is illustrated in Figure 2c. This resistance is frequently linked to the overexpression of P-glycoprotein (P-gp). P-gp actively expels drugs from inside the cells through the “extracellular substrate drainage pump”, which reduces the effectiveness of chemotherapy [39]. To overcome this challenge, the development of potent P-gp inhibitors has emerged as a crucial therapeutic strategy. By blocking P-gp efflux activity, these inhibitors can restore drug sensitivity in resistant tumors, thereby improving treatment outcomes. A study by Feng et al. [40] synthesized flavonoid compound **13** (Figure 3), which significantly increased the concentration of paclitaxel in tumors by inhibiting P-gp activity.

### 2.4. Induction of Cell Cycle Arrest

The mechanism of cell cycle arrest is illustrated in Figure 2d. The cell cycle—comprising the G1, S, G2, and M phases—is tightly regulated by cyclins, Cyclin-Dependent Kinases (CDKs), and their inhibitors (CKIs) [41]. Specifically, the G1/S transition is driven by the Cyclin D1-CDK4/6 complex, which phosphorylates the retinoblastoma protein (pRb) to promote S-phase entry. Cyclin A associates with CDK2 to ensure the completion of DNA replication and the transition into the G2 phase. Cyclin B partners with CDK1 to push cells into the M phase. Cyclin-dependent Kinase Inhibitor 1 (p21), which acts as a downstream effector of p53, can inhibit the activity of Cyclin-CDK complexes, leading to cell cycle arrest. The development of anti-tumor drugs aimed at inducing tumor cell cycle arrest primarily focuses on targeting Cyclin-CDK complexes and the p53-p21 signaling pathway [42,43]. Compound **11** (Figure 3) was synthesized by Bak et al. [36]. In NCI-H460 cells, compound **11** blocked the cell cycle in the sub-G1 phase by downregulating the expression of Cyclin A and Cyclin D1 and downregulating the level of phospho-pRb. In A549 cells, compound **11** also down-regulated Cyclin D1 and phospho-pRb expression. It inhibits the activity of CDK2 and CDK4/6 by upregulating the level of p21/p27, resulting in G0/G1 phase cell cycle arrest. Additionally, Peng et al. [44] developed compound **14** (Figure 3), which induces G1 phase 45 arrest in specific cell lines by upregulating the levels of p21 and p27. This compound inhibited the formation of the Cyclin D1/CDK4 complex and blocked the phosphorylation of Rb.

### 2.5. Inhibiting Tumor Cell Angiogenesis

The mechanism of inhibiting tumor cell angiogenesis is illustrated in Figure 2e. When the volume of a solid tumor exceeds a critical threshold, it must meet its proliferation demands by forming new blood vessels. This pathological process is primarily driven by a cascade of reactions involving the VEGF and its receptor (VEGFR) family. Therefore, targeted inhibition of pathological angiogenesis has become an essential strategy for anti-tumor therapy [45,46]. Jiang et al. [47] designed and synthesized flavonoid compound **15** (Figure 3), which exhibited significant anti-tumor activity. In vitro experiments demonstrated that the IC_50_ of this compound on A549 cells was 4.73 μM. In a zebrafish embryo angiogenesis model, its inhibitory effect at a concentration of 2.5 μM was comparable to that of a classic VEGFR inhibitor (SU5416). In addition, in vitro studies have shown that the compound can inhibit angiogenesis by reducing the proliferation, migration, and tube formation of human umbilical vein endothelial cells.

### 2.6. Inhibiting EGFR Kinase Activity

The mechanism by which epidermal growth factor receptor (EGFR) kinase activity is inhibited is illustrated in Figure 2e. The EGFR gene mutations occur frequently in NSCLC. Abnormal activation of the EGFR tyrosine kinase domain activates downstream signaling pathways, including the rat sarcoma virus oncogene homolog (RAS)/rapidly accelerated fibrosarcoma (RAF)/MAPK/ERK kinase (MEK)/extracellular signal-regulated kinase (ERK) and phosphatidylinositol 3-kinase (PI3K)/protein kinase B (AKT)/mTOR pathways, leading to the malignant proliferation of tumor cells. To address this, tyrosine kinase inhibitors (TKIs) were developed. These drugs competitively inhibited the ATP binding domain of EGFR, effectively blocking the abnormal signal transduction, and so have been used in the first-line treatment of EGFR-mutated NSCLC [48]. Wang et al. [49] synthesized flavonoid compound **16** (Figure 3). The combination of this compound with cisplatin produced a synergistic anti-tumor effect, significantly reducing the phosphorylation levels of EGFR and its downstream proteins AKT and ERK.

## 3. Structure–activity Relationship of Flavonoid Derivatives

### 3.1. C-7 Substituted

#### 3.1.1. Functional Group Substitution

**Figure 4:**
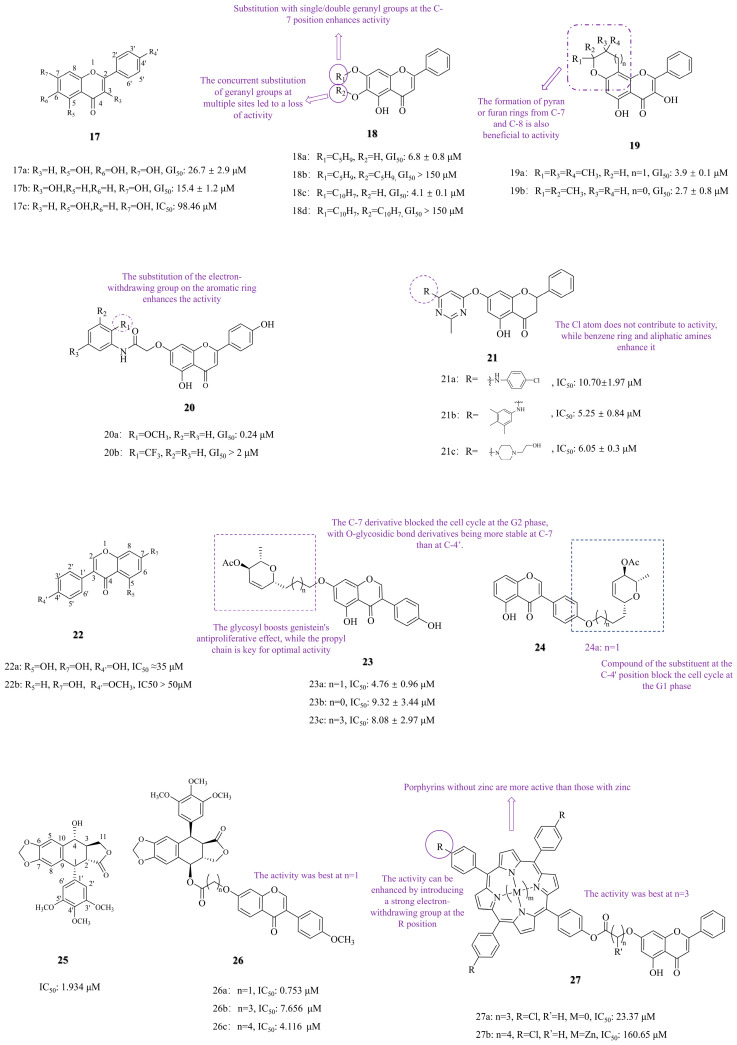
C-7 substituted.

Studies have shown that 5,6,7-trihydroxyflavone (baicalein, compound **17a**, Figure 4) exerts a dual anti-tumor effect by inducing apoptosis and autophagy in A549 lung cancer cells, and 3,7-dihydroxyflavone (compound **17b**, Figure 4) inhibits tumor growth through a pro-apoptotic mechanism [50,51]. Studies have indicated that the geranyl groups of flavonoids can enhance their growth-inhibitory effects on colon and breast cancer cell lines [52,53]. Neves MP et al. [54] investigated the anti-tumor activity of compounds by introducing a geranyl group at the C-7 position of compounds **17a** and **17b**. The SAR revealed that compounds with single or double geranyl groups, compound **18a** (Figure 4, GI_50_: 6.8 ± 0.8 μM) and compound **18c** (Figure 4, GI_50_: 4.1 ± 0.1 μM), demonstrated significantly enhanced activity against NCI-H460 lung cancer cells compared to baicalein (GI_50_ = 26.7 ± 2.9 μM). Similarly, pyran/furan-containing analogs **19a** (GI_50_: 3.9 ± 0.1 μM) and **19b** (GI_50_: 2.7 ± 0.8 μM) exhibited significantly enhanced potency compared to baicalein, with **19b** showing GI_50_ value that was 10 times lower against NCI-H460 lung cancer cells. Conversely, compounds with multi-substituted isoprene chains or those with longer chains (compounds **18b** and **18d**, Figure 4) showed a loss of activity (GI_50_ > 150 μM) against Ncl-H460 lung cancer cells.

Mayer et al. [55] synthesized 7-aminochrysin derivatives (17c, Figure 4) from 5,7-dihydroxyflavone (chrysin). The SAR analysis revealed that introducing a carbamoylmethoxy group at the C-7 position of compound **20a** (Figure 4, GI_50_: 0.24 μM), which carries a methoxy group on the flavonoid scaffold, significantly enhanced its activity against NCI-H522 lung cancer cells compared to chrysin (IC_50_: 98.46 μM). In contrast, electron-withdrawing substitution on the aromatic ring of compound **20b** (Figure 4) resulted in a loss of anti-lung cancer activity, demonstrating negligible potency (GI_50_ > 2 μM) against a panel of NSCLC cell lines (A549, EKVX, HOP-62, HOP-92, NCI-H226, NCI-H23, NCI-H322M, and NCI-H460). Additionally, Yu et al. [56] connected chrysin with pyrimidine groups. The SAR revealed that while chlorine-substituted compound **21a** (IC_50_: 10.70 ± 1.97, Figure 4) had weak activity, substitutions with a benzene ring-containing compound **21b** (IC_50_: 5.25 ± 0.84 μM, Figure 4) and aliphatic amine-containing compound **21c** (IC_50_: 6.05 ± 0.3 μM, Figure 4) showed significantly improved potency against A549 cells. Their efficacy surpassed that of the reference drug erlotinib (IC_50_: 18.09 ± 1.71 μM).

Studies have indicated that 4′,5,7-Trihydroxyisoflavone (genistein, compound **22a**, Figure 4) shows its effect by inhibiting the stemness of lung cancer cells and inducing apoptosis [57,58]. Glycosylation modifications have been shown to enhance drug targeting and improve pharmacokinetic characteristics [59,60]. Based on this, Rusin et al. [61] synthesized some derivatives by glycosylation at C-7 of compound **22a** (Figure 4). The SAR showed that linker length and glycosyl group jointly determine activity. The inhibitory activities of compounds **23a** (IC_50_: 4.76 ± 0.96 μM, Figure 4), **23b** (IC_50_: 9.32 ± 3.44 μM, Figure 4), and **23c** (IC_50_: 8.08 ± 2.97 μM, Figure 4) against A549 cells demonstrate that the propyl linker confers the highest potency. All of these lipophilic glycoside-containing compounds exhibited significantly higher activity compared to compound **22a** (IC_50_ ≈ 35 μM, Figure 4). Additionally, Papaj et al. [29] studied the bioavailability of these derivatives and discovered that glycosyl conjugates metabolized more slowly in vivo than genistein. Mechanistic studies revealed that the C-7-substituted compound **23a** (Figure 4) blocked cell mitosis at the G2 phase, while the C-4′-substituted compound **24a** (Figure 4) blocked the G1 phase.

Molecular hybridization was accomplished by merging different pharmacophores to create hybrid compounds with enhanced activity [62]. Studies have shown that 7-Hydroxy-4′-methoxyisoflavone (formononetin, compound **22b**, Figure 4) and podophyllotoxin (compound **25**, Figure 4) both had anti-lung cancer activity [63,64,65]. Yang et al. [66] designed and synthesized new hybrid molecules by connecting the C-7 position of compound **22b** to the C-4 position of compound **25**. The SAR revealed that the compound with the shortest carbon chain, compound **26a** (Figure 4, IC_50_: 0.753 μM), exhibited the most vigorous activity against A549 cells, surpassing the activity of the two individual monomers. Additionally, compound **26a** induced apoptosis in A549 cells by activating the caspase pathway.

#### 3.1.2. Photosensitizer Conjugation and Photodynamic Therapy

Porphyrin compounds are widely used in photodynamic therapy (PDT) due to their strong photosensitizing properties and selective accumulation in tumor tissues via the enhanced permeability and retention (EPR) effect [67,68]. For instance, Zhang et al. [28] designed a porphyrin-chrysin conjugate (compound **27a**, Figure 4) that exhibits potent anti-tumor activity through PDT-mediated ROS generation. The SAR revealed that the non-zinc compound **27a** (IC_50_: 23.37 μM) showed significantly higher efficacy against A549 cells than its zinc-containing compound **27b** (IC_50_: 160.65 μM), likely due to the inhibitory effect of zinc ions on ROS generation. Notably, 27a outperformed the positive control 5-fluorouracil (IC_50_: 79.56 μM), emphasizing its therapeutic potential.

### 3.2. C-6 Substituted

**Figure 5:**
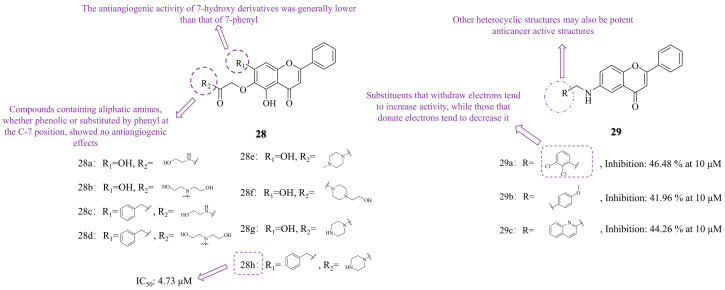
C-6 substituted.

Studies have shown that baicalein (compound **17a**, Figure 4) inhibits the development of tumor vasculature by disrupting tumor angiogenesis [69]. However, there have been few studies on baicalein derivatives in the direction of angiogenesis inhibition, and baicalein has low water solubility. Adding hydrophilic groups to its molecular structure can improve its solubility and oral bioavailability [70]. Based on this, Jiang et al. [47] introduced hydrophilic groups and nitrogen-containing heterocycles at the 6-position of baicalein to develop novel anticancer agents and to explore the effects of C-6/C-7 substituents of baicalein on its antiangiogenic activity. The SAR revealed that the antiangiogenic activity of 7-hydroxy derivatives was generally lower than that of 7-benzyl derivatives. Neither phenolic nor benzyl substitutions at C-7 could rescue the loss of antiangiogenic activity induced by C-6 aliphatic amine derivatives (compounds **28a~d**, Figure 5), which exhibited no antiangiogenic effect. Compound **28g**, substituted by the piperazine terminal group, retained antiangiogenic activity. In contrast, piperazine *N*-terminal-substituted compounds, such as **28e** (*N*-methylpiperazine) and **28f** (*N*-ethylpiperazine), lost their activity. Notably, compound **28h**, bearing a piperazine acetamide group at the 6-position, demonstrated the best antiangiogenic activity, along with potent anti-tumor effects against A549 cells (IC_50_ = 4.73 μM).

Thorat NM et al. [71] synthesized a series of *N*-benzyl derivatives of 6-amino flavones (compounds **29a~c**, Figure 5). The SAR revealed that electron-withdrawing groups (such as chlorine, bromine, and cyano) on the aniline ring enhanced activity, with compound **29a** (2,3-dichloro) exhibiting the highest activity (inhibition rate: 46.48% at 10 μM) against A549 lung cancer cells, surpassing doxorubicin (inhibition rate: 43.76% at 10 μM). Conversely, electron-donating groups (such as methoxy and ethoxy) resulted in lower activity, with compound **29b** showing an inhibition rate of 41.96% at 10 μM against A549 lung cancer cells. Additionally, heterocyclic substituents, such as the 2-methylquinoline in compound **29c**, exhibited good activity, with an inhibition rate of 44.26% at 10 μM against A549 lung cancer cells.

### 3.3. Other Modifications

#### 3.3.1. Functional Group Substitution

**Figure 6:**
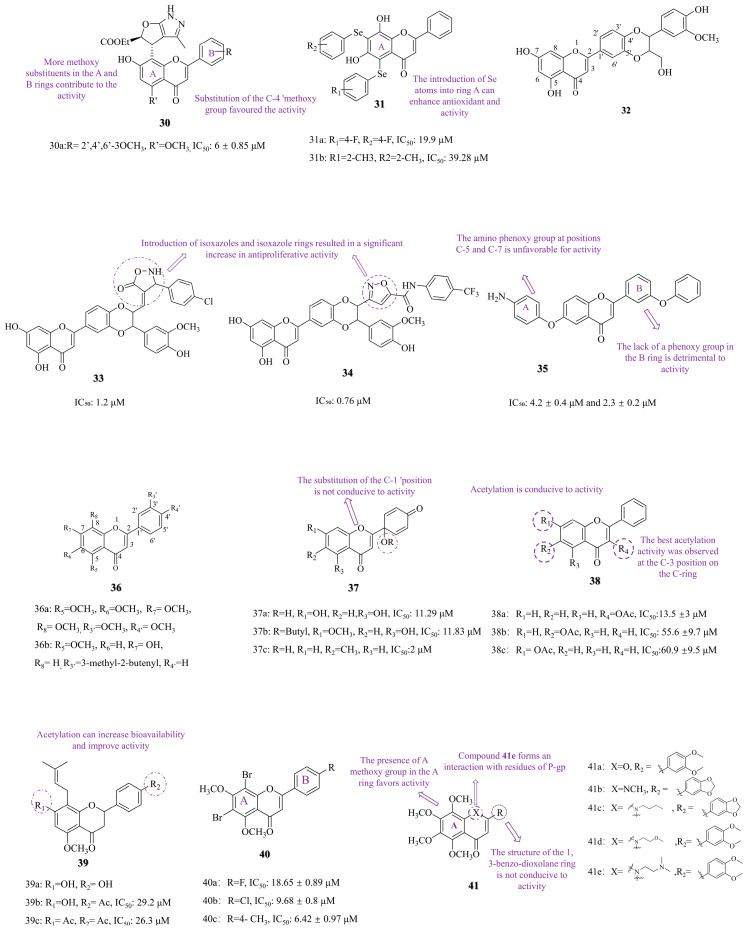
Other modifications.

Tangeti vs. et al. [72] introduced the furan-pyrazole structure into 7-hydroxyflavone and then added methoxy groups to the rings of these compounds A and B. The SAR revealed that adding more methoxy groups to these rings enhanced their activity, with the C4′-methoxy substitution particularly crucial for effectiveness. The compound **30a** (Figure 6, IC_50_: 6 ± 0.85 μM), which features four methoxy substituents on the A and B rings, displayed the most potent antiproliferative activity against A549 lung cancer cells. This activity was comparable to that of the positive control drugs doxorubicin (IC_50_: 0.65 ± 0.04 μM) and paclitaxel (IC_50_: 0.175 ± 0.01 μM) against A549 lung cancer cells.

Selenium (Se), a [73,74] redox-regulating trace element, has shown potential in chemotherapy [73,74]. Fonseca SF et al. [75] hybridized Se atoms with 5,7-dihydroxyflavone (chrysin, compound **17c**, Figure 4) to create novel compounds. The SAR revealed that adding Se atoms to the A-ring improved antioxidant and anticancer activities. Compound **31a** (Figure 6, IC_50_: 19.9 µM) exhibited the most potent anticancer effect against A549 lung cancer cells after 72 h, while compound **31b** (Figure 6) showed the highest antioxidant activity. Although the in vitro anti-tumor activity of target compound **31a** was slightly lower than that of the positive control cisplatin (IC_50_: 5.32 μM, A549, 72 h), its unique selenoether structure and straightforward synthetic route suggest a potentially distinct mechanism of action.

The introduction of isoxazole into drug structures has been shown to induce apoptosis in tumor cells [76]. Compound **32** (Figure 6), known as hydnocarpin, is a natural flavonoid lignan that induces apoptosis in lung cancer cells. Arya JS et al. [77] synthesized a series of isoxazole and isoxazolone compounds based on compound **32** and studied their impact on lung cancer cells. The SAR revealed that incorporating these heterocycles into the structure significantly improved antiproliferative activity. Compound **34** (Figure 6, IC_50_: 0.76 μM) exhibited potent activity against A549 lung cancer cells after 48 h, outperforming Compound **33** (Figure 6, IC_50_: 1.2 μM). Mechanistic studies demonstrated that both compounds induce tumor cell death via the caspase-dependent apoptosis pathway and increase ROS levels in a dose-dependent manner, activating the mitochondrial apoptotic pathway.

Mobbili et al. [78] synthesized amino phenoxy flavonoid derivatives and studied their effects on lung cancer. The SAR revealed that the activity of compounds with the aminophenoxy group on the A ring and the phenoxy group on the B ring was enhanced. In contrast, compounds lacking a phenoxy group or having an aminophenoxy group on the C-5 or C-7 A ring are less effective. Compound **35** (Figure 6) showed significant activity against A549 and H1975 lung cancer cell lines, with IC_50_ values of 4.2 ± 0.4 μM and 2.3 ± 0.2 μM, respectively, which were comparable to the activity of the control drug doxorubicin against these two cancer cells, which had IC_50_ values of 0.2 μM and 2.5 μM, respectively. Mechanism studies have shown that compound 35 induces apoptosis and G2/M phase cell cycle arrest by upregulating p21 expression.

Protoapigenone (PA, Compound **37a**, Figure 6) is an oxidized derivative of the common flavonoid apigenin with significant anti-tumor activity [79,80]. Danko B et al. [81] synthesized various 1′-O-alkyl derivatives with tiny pores. The SAR revealed that C-1′-hydroxyl derivatives, especially compound **37c** (Figure 6, IC_50_: 2 μM), exhibited potent activity against A549 lung cancer cells, and their activity was much higher than that of the PA (IC_50_: 11.29 μM). However, replacing the C-1′ hydroxy group reduced the activity, as shown in compound **37b** (Figure 6, IC_50_: 11.83 μM).

Stompor et al. [82] enhanced the antiproliferative activity of compounds by acetylating the hydroxyl group of flavonoids. The SAR revealed that acetylated compounds were more effective than their non-acetylated versions, primarily when acetylated at the C-3 position of the C-ring (compound **38a**, Figure 6, IC_50_: 13.5 ±3 μM), which had more significant activity than those acetylated at the C-6 and C-7 positions (compounds **38b**, IC_50_: 55.6 ± 9.7 μM and **38c**, IC_50_: 60.9 ± 9.5 μM, Figure 6), but their activity is not as great as the positive control drug cisplatin (IC_50_: 6.8 ± 0.8 μM). Isoxanthohumol (compound **39a**, Figure 6), a flavonoid derived from hops and beer, was investigated by Stompor et al. [83]. Acetylation at the C-4′ and C-7 positions yielded compounds (**39b~39c**, Figure 6). The SAR study demonstrated that acetylation increased the water solubility and bioavailability. Acetylated compounds **39b** (IC_50_: 29.2 μM) and **39c** (IC_50_: 26.3 μM) had lower toxicity to normal cells, but their anticancer activity against A549 lung cancer cells was still lower than that of the positive control cisplatin (IC_50_: 6.8 μM).

The incorporation of bromine atoms into drug structures enhances therapeutic efficacy by forming halogen bonds with proteins, which increases selectivity for active sites and stabilizes drug–protein complexes [84]. Nkoana JK et al. [30] synthesized 3,5-dibromo-4,6-dimethoxyflavone derivatives (Compounds **40a~c**, Figure 6) by substituting the C-4′ position of flavonoids. These derivatives showed IC_50_ values of 18.65 ± 0.89 µM; 9.68 ± 0.8 µM; 6.42 ± 0.97 μM against A549 lung cancer cells. The SAR showed that substitutions on the B ring (Cl or methyl) are beneficial to activity, and the methoxy group on the A ring is also key to optimizing activity. That series of compounds exhibited strong activity against the A549 cells compared to the analogous quercetin (IC_50_: 35.38 ± 1.78 μM), although it was moderate compared to nintedanib (IC_50_: 0.74 ± 0.15 μM).

#### 3.3.2. Heterocyclization

Feng et al. [40] synthesized derivatives of 3′,4′,5,6,7,8-hexamethoxyflavone (Nobiletin, **41a**, Figure 6). The SAR revealed that increasing the number of methoxy groups on the A-ring enhanced the inhibitory activity. However, placing a methoxy group at the C-5 position reduced this activity due to steric hindrance. Additionally, substituting the benzene ring of the B-ring with a 1,3-benzodioxole structure (compounds **41b** and **41c**, Figure 6) was also detrimental to activity, again due to steric hindrance. In the X-position modifications, compound **41e** (Figure 6) could interact with P-gp through hydrophobic and van der Waals interactions. In the A549/T xenograft model, the combination of compound **41e** (50 mg/kg) with paclitaxel (15 mg/kg) reduced tumor volume by 57%, demonstrating superior efficacy to Nobiletin.

### 3.4. The SARs Summary of Flavonoid Derivatives in Anti-Lung Cancer Activity

Flavonoid derivatives exhibit promising prospects as anti-lung cancer agents due to their multi-target effects and high structural modifiability (Table 1). Through SAR analysis, the following key structural modification patterns were elucidated: C-7 modifications, including geranylation **18c** and glycosylation **23a**, and pyran/furan analogs **19a** and **19b**, significantly enhance bioactivity. While molecular hybridization at position **26a** demonstrates synergistic effects surpassing monomeric drug limitations. The trimethoxyphenyl ring of **26a** precisely occupied the colchicine-binding pocket of the β-subunit, while the methoxylated isoflavone ring was positioned at the αβ-subunit interface region, forming a hydrogen bond with the LEU-248 residue of the β-subunit. C-6 substitutions demonstrate specialized applications in antiangiogenesis, with the best-performing compound, **28h**, featuring a piperazine acetamide group. In contrast, C-7 substitutions consistently yielded superior potency, as evidenced by the lower IC_50_ and GI_50_ values. The introduction of electron-withdrawing groups such as halogens at both sites is conducive to activity. The most potent C-7 derivative, **20a**, with a carbamoylmethoxy group, demonstrated exceptional activity with a GI_50_ of 0.24 μM. Furthermore, heterocyclization strategies (isoxazole and isoxazolone compounds), such as compounds **33** and **34**, markedly improve anti-tumor efficacy. Molecular docking analysis revealed that compound **34** had different binding modes with the anti-apoptotic protein Bcl-xL (PDB: 1OXN and 1OXQ). In the 1OXN complex, compound **34** formed hydrogen bonds with Tyr128 and Arg123 residues while simultaneously engaging in π–π stacking interactions with Phe81 and Tyr128. In the 1OXQ complex, the compound exhibited a hydrogen bond with Asp96 and π–π stacking interactions with His118 and Phe81. Compound **33** only had hydrogen bond interaction with ala80 and had low inhibitory activity. These findings underscore the therapeutic potential of tailored flavonoid-based scaffolds in lung cancer treatment.

## 4. Conclusions and Future Perspectives

NSCLC is one of the leading causes of cancer-related deaths worldwide. As natural compounds, flavonoids demonstrate significant anti-tumor activity and serve as essential scaffolds for developing anticancer drugs. To optimize their efficacy and drug-like properties, structural modifications—such as functional group substitution, halogenation, and heterocyclization—have been employed, enabling these derivatives to target tumors through diverse mechanisms.

To accelerate discovery, multidisciplinary approaches integrating computer-aided drug design (CADD) are critical [85]. CADD leverages computational modeling, bioinformatics, and quantum chemical calculations to streamline drug discovery and development processes. By integrating computational approaches with structural biology and molecular dynamics, CADD enables high-throughput virtual screening, binding affinity prediction, and rational drug optimization—significantly enhancing research efficiency [86]. This approach facilitates the identification of promising small molecule candidates from structural databases (e.g., ZINC) for conjugation with flavonoid scaffolds. Subsequent molecular docking simulations against critical NSCLC targets, including EGFR, ALK, PD-L1, and PI3K/AKT/mTOR pathway proteins, enable the design of novel therapeutic compounds with optimized binding characteristics.

Flavonoid derivatives can be further optimized into peptide–drug conjugates (PDCs) to enhance tumor selectivity [87]. Peptides can serve as targeted transport in the body, which can be applied to cancer medication in the target. Peptide–drug conjugates consist of three parts in synergy, namely, a homing peptide, a linker, and a cytotoxic payload, which ensure arrival at the selected receptor and exert pharmacological effect [88]. With two PDCs already approved by the FDA [89,90], this approach shows significant potential [88,91].

Finally, nanotechnology provides a complementary strategy to improve flavonoid delivery. For instance, encapsulating flavonoid derivatives in nanocarriers—such as metal–organic frameworks (MOFs), which exhibit tunable porosity and high drug loading capacity—can enhance tumor-targeted delivery, therapeutic precision, and controlled release by mitigating premature degradation [92]. The nanocarrier is a novel carrier system for drug delivery composed of a nanosized structure ranging from 1 to 500 nm in size [93]. There are other systems used for drug delivery or other aspects of clinical diagnosis and treatment, including liposomes, dendrimers, nanoshells, and metal–organic frameworks [92,94]. The combination of flavonoids with nanocarriers, also known as flavonoid-based nanocarriers, is an efficient method to overcome major limitations, such as low aqueous solubility, poor bioavailability, and low stability in the stomach [95]. Additionally, developing solvent-free, catalytically efficient methods for synthesizing flavonoid backbones can help minimize adverse effects on the environment [96].

To maximize clinical potential, future efforts should integrate sustainable synthesis methods, computational optimization, and targeted nanoformulations, ultimately paving the way for more effective and environmentally conscious NSCLC therapies.

## Figures and Tables

**Table 1:** The most active flavonoid derivative molecules and their anti-lung cancer activities.

Modification	Compound ID	Activity	Cells Line	Reference
C-7 substituted	**18c**	4.1 ± 0.1 μM	NCI-H460	[54]
	**19a**	3.9 ± 0.1 μM		
	**19b**	2.7 ± 0.8 μM		
	**20a**	0.24 μM	NCI-H522	[55]
	**23a**	4.76 ± 0.96 μM	A549	[61]
	**26a**	0.753 μM	A549	[66]
C-6 substituted	**28h**	4.73 μM	A549	[47]
Other modifications	**33**	1.2 μM	A549	[77]
	**34**	0.76 μM		
	**35**	0.4 μM 2.3 ± 0.2 μM	A549	[78]
			H1975	
	**37c**	2 μM	A549	[81]

## Data Availability

Data sharing is not applicable to this article.
